# Upcycling the Spent Mushroom Substrate of the Grey Oyster Mushroom *Pleurotus pulmonarius* as a Source of Lignocellulolytic Enzymes for Palm Oil Mill Effluent Hydrolysis

**DOI:** 10.4014/jmb.2103.03020

**Published:** 2021-04-29

**Authors:** Nurul Anisa Mat Yunan, Tan Yee Shin, Vikineswary Sabaratnam

**Affiliations:** 1 Mushroom Research Centre, Universiti Malaya, 50603 Kuala Lumpur, Malaysia; 2 Institute of Biological Sciences, Faculty of Science, Universiti Malaya, 50603 Kuala Lumpur, Malaysia

**Keywords:** *Pleurotus pulmonarius*, edible mushroom, enzymatic hydrolysis, palm oil mill effluent, spent mushroom substrate, biohydrogen

## Abstract

Mushroom cultivation along with the palm oil industry in Malaysia have contributed to large volumes of accumulated lignocellulosic residues that cause serious environmental pollution when these agroresidues are burned. In this study, we illustrated the utilization of lignocellulolytic enzymes from the spent mushroom substrate of *Pleurotus pulmonarius* for the hydrolysis of palm oil mill effluent (POME). The hydrolysate was used for the production of biohydrogen gas and enzyme assays were carried out to determine the productivities/activities of lignin peroxidase, laccase, xylanase, endoglucanase and β-glucosidase in spent mushroom substrate. Further, the enzyme cocktails were concentrated for the hydrolysis of POME. Central composite design of response surface methodology was performed to examine the effects of enzyme loading, incubation time and pH on the reducing sugar yield. Productivities of the enzymes for xylanase, laccase, endoglucanase, lignin peroxidase and β-glucosidase were 2.3, 4.1, 14.6, 214.1, and 915.4 U g^-1^, respectively. A maximum of 3.75 g/l of reducing sugar was obtained under optimized conditions of 15 h incubation time with 10% enzyme loading (v/v) at a pH of 4.8, which was consistent with the predicted reducing sugar concentration (3.76 g/l). The biohydrogen cumulative volume (302.78 ml H_2_.L^-1^ POME) and 83.52% biohydrogen gas were recorded using batch fermentation which indicated that the enzymes of spent mushroom substrate can be utilized for hydrolysis of POME.

## Introduction

*Pleurotus pulmonarius* (Fries) Quélet is one of the top commercially cultivated mushrooms in the world due to its wide adaptability to the natural environment and ability to utilize a range of agroresidues as substrate by secreting a broad array of ligninolytic and cellulolytic enzymes [1]. However, the industry has struggled to manage and discard the spent mushroom substrate (SMS). Singh *et al*. [2] reported that around 800 g of SMS is discarded as waste for every 200 g of mushroom produced. The inefficient practices of disposal and management of the wastes has spurred studies on utilizing the biomass for production of value-added products [3].

SMS contains high organic matter, mineral nutrients and enzymes [4]. The common ways of recycling SMS include transformation into biofuel [5], biofertilizer [6-8] and reducing sugar for the fermentation process [9, 10]. The study showed that SMS pretreated with dilute acid and alkaline reagents could improve cellulose degradation into sugar. Thus, SMS extract could be used as a potential feedstock for reducing sugar production and minimize environmental problems. Currently, there are several reports on the applications of enzymes from SMS [11-13].

Thus, SMS could be a potential source of lignocellulolytic enzymes to hydrolyze palm oil mill effluent (POME) which has been classified as one of the major causes of water pollution [14]. Raw POME is produced from a combination of sterilizer condensate, separator sludge and hydrocyclone wastewater in a proportion of 9:15:1, respectively [15].

Conversion of POME into value-added products has been identified as an alternative method of waste treatment. Moreover, POME is recognized as one of the lignocellulosic biomass substrates in the biofuel industry for conversion into fermentable sugars [16]. The sugars can be utilized for a higher value feedstock to produce biogas and biofuel. High-cost commercial enzymes have been used for POME hydrolysis which converts the organic substances in POME into monomeric sugars [17-19]. However, studies on the potential use of enzymes from SMS for the hydrolysis of POME have not been explored. Further, the high concentrations of lipid, minerals, carbohydrates, protein, and nitrogenous compounds in POME make it a suitable substrate for biohydrogen production [20].

Hydrogen production from biomass has been a captivating substitute for fossil fuels while hydrogen is known as the cleanest renewable energy source with zero carbon emission [21]. Many studies have been conducted on the biohydrogen production from POME involving different experimental conditions such as pre-treatment of POME using *Clostridium butyricum* [22], application of a granular sludge system and fixed film reactor [23], and two-stage thermophilic and mesophilic fermentation condition [24].

Hence, the aim of this study was to hydrolyze POME using enzymes extracted from the SMS of *P. pulmonarius*. Three factors that might affect the hydrolysis rate (*viz*. incubation time, enzyme loading and pH) were analyzed to examine the optimum conditions to hydrolyze lignocellulolytic components in POME into simple sugar. A central composite design (CCD) in response surface methodology (RSM) was chosen for the optimization of hydrolysis [25]. Finally, a validation batch study to examine the feasibility of utilizing the hydrolysate for biohydrogen production was conducted.

## Materials and Methods

### Enzymes Extraction

*P. pulmonarius* mushroom substrate bags were collected from Nas Agro Farm in Sepang, Selangor, Malaysia. Initially, 11-week-old inoculated substrate bags were randomly collected. The substrates of five bags were mixed manually. Then, three replicate samples were randomly chosen for extraction. The extraction was conducted by adding 20 g of SMS to 100 ml of tap water (pH 4.0) in a 250 ml Erlenmeyer flask [2]. The mixture was incubated in a shaking incubator (Daihan Labtech Co., Singapore) with a speed of 150 rpm for one hour at 4°C. The supernatant of crude enzymes was isolated from the solids by centrifugation at 9,000 ×*g* for 20 min and stored at -20°C before enzyme assays. The crude enzymes were concentrated using a freeze dryer (Alpha 1-4 LDplus, Christ) and the concentration was fixed at 100 mg/ml for hydrolysis of POME. The enzyme powder was placed in a sealed container and stored in a 4°C fridge before further hydrolysis. Productivities of the concentrated enzymes were measured and reported as U/g of the substrate [2].

All the enzyme activities in this study were measured by a UV-160A spectrophotometer (Shimadzu). The enzyme activity unit (U) was defined as the amount of enzyme required to produce 1 μmole of product/min. Meanwhile for laccase, enzyme activity unit (U) was defined as the amount of enzyme producing one-unit change in absorbance/min. The result of this study was reported in terms of productivity based on the number of units obtained per gram (U/g) of the SMS used [2].

### Measurement of SMS Enzyme Activity

Lignin peroxidase (LiP) activity was determined using the method described by Have *et al*. [26]. The reaction mixture consisted of 0.2 ml crude enzyme, 2.4 ml of 100 mM sodium tartrate buffer (pH 3.0) and 0.2 ml of 2 mM veratryl alcohol. Veratraldehyde was used as the standard. The experiment was started by adding 0.2 ml of freshly prepared 0.5 mM H_2_O_2_ (final concentration). The absorbance reading was recorded at 310 nm after 5 min of incubation.

Laccase assay was measured using the method as described by Harkin and Obst; Leonowicz and Grzywnowicz [27, 28]. The reaction mixture contained 0.5 ml of the crude enzyme, 3.0 ml of sodium citrate buffer (pH 4.8) and the substrate used was 0.5 ml of 0.1 mM syringaldazine in ethanol (50%, w/v). The activity of laccase was examined by recording the absorbance at 525 nm. The quantity of enzyme releasing one unit of absorbance change min^-1^ g^-1^ of the substrate represents one unit of activity. Xylanase activity was measured using the method of Bailey *et al*. [29]. The reaction mixture consisted of 0.2 ml of crude enzyme and 1.8 ml of substrate. The solution was mixed and incubated at 40°C for 1 h in water bath with moderate shaking. The amount of sugars liberated was quantified by dinitrosalicylic acid (DNS) method [30]. Xylose was used as the standard. The activity was defined as the number of xylanases needed to produce 1 μmole of xylose min^-1^ at a wavelength of λ=575 nm. Endoglucanase activity was measured followed the method by Kim *et al*. [31]. Sodium salt of carboxymethyl cellulose solution with medium viscosity (1% w/v) was used as the substrate. Glucose was used as the standard. The reaction comprised 1.8 ml of substrate and 0.2 ml of the crude enzyme. The solution was agitated adequately in a water bath and incubated at 40°C for 30 minutes with gentle agitation. The reducing sugar liberated was quantified using the DNS method [30]. The activity was defined as the quantity of endoglucanase required to produce 1 μmole of glucose min^-1^ at a wavelength of λ=575 nm. β-glucosidase activity was determined following the method described by Kim *et al*. [31]. The substrate used was 0.5 mM p-nitrophenyl-β-D-glucopyranoside in 50 mM sodium citrate buffer (pH 4.8). The reaction mixture consists of 1.8 ml of substrate and 0.2 ml of crude enzyme. Each of the test tubes was protected using aluminium paper since the released p-nitrophenol is photosensitive. The solution was incubated at 40°C for 30 min in a water bath with gentle agitation. The incubation was stopped by the addition of 2.0 ml of 1 M Na_2_CO_3_. The amount of p-nitrophenol liberated was calculated using p-nitrophenol as the standard. The activity was defined as the number of β-glucosidases needed to produce 1 μmole of p-nitrophenol min^-1^ g^-1^ of the substrate at a wavelength of λ=400 nm.

### Statistical Analysis

Analysis of variance (ANOVA) was done with means of triplicate values of enzyme productivities and Duncan’s Multiple Range Test was employed to determine significant differences between the three means at 95% least significant difference (*p* < 0.05).

### Characteristics of the Palm Oil Mill Effluent

POME was collected from Jugra Palm Oil Mill in Banting, Selangor, Malaysia after the acidification process [32]. The POME was set aside at room temperature for 20 min to settle the solids and the supernatant was extracted and used as pre-settled POME [25]. Before enzymatic hydrolysis, the pre-settled POME was preserved at 4°C in a cold room. The pH of the pre-settled POME was 4.99, with chemical oxygen demand of 27,000-28,000 mg/l. The total nitrogen and protein recorded were 0.11% and 0.70%, respectively. Additionally, the concentration of the reducing sugar in POME was recorded at 0.77 g/l. Meanwhile, POME sludge collected from an anaerobic pond of the same palm oil mill was used as inoculum for biohydrogen production [32].

### Experimental Design for Optimization

The POME hydrolysis was carried out in an Erlenmeyer flask (250 ml) with a screw cap. The mixture consists of 10% (v/v) concentrated enzyme loading (10 ml enzyme/100 ml of POME). The preliminary experiments (unpublished data) showed that enzymes from SMS were able to hydrolyze POME. Thus, Design-Expert software (Stat-Ease Inc., USA; Version 6.0.7) was used for the optimization of hydrolysis in terms of experimental design, data analysis, and graphical study of the data. Overall 20 experiments were conducted to optimize the process parameters. The concentrated enzymes (0%, 5%, 10%) were inoculated into the POME in the rotary shaker under different incubation times (0, 12, 24 h). The mixture was adjusted to different conditions of pH value (4.8, 5.4, 6.0) with 1 N HCL or 1 N NaOH [26]. The significance of the three factors was determined by the ANOVA. Equation (1) was used to obtain the coefficients of the polynomial model:

$$Y=\beta_{0}+\beta_{i}x_{i}+\beta_{j}x_{j}+\beta_{ii}x_{i}^{2}+\beta_{jj}x_{j}^{2}+\beta_{ij}x_{i}x_{j}+\ldots$$ (1)

where Y is the predicted response for reducing sugar yield, *β* is the regression, i is linear and j is a quadratic coefficient, respectively. A 3D surface plot displayed the effect of the independent factors on the response. The significance of the second-order equation model was measured by a significant *F*-value and an insignificant lack-of-fit *F*-value. The estimated optimum value was verified by an analysis using the chosen optimum value of the three factors.

### Batch Fermentation for Biohydrogen Production

**Fig. 1:**
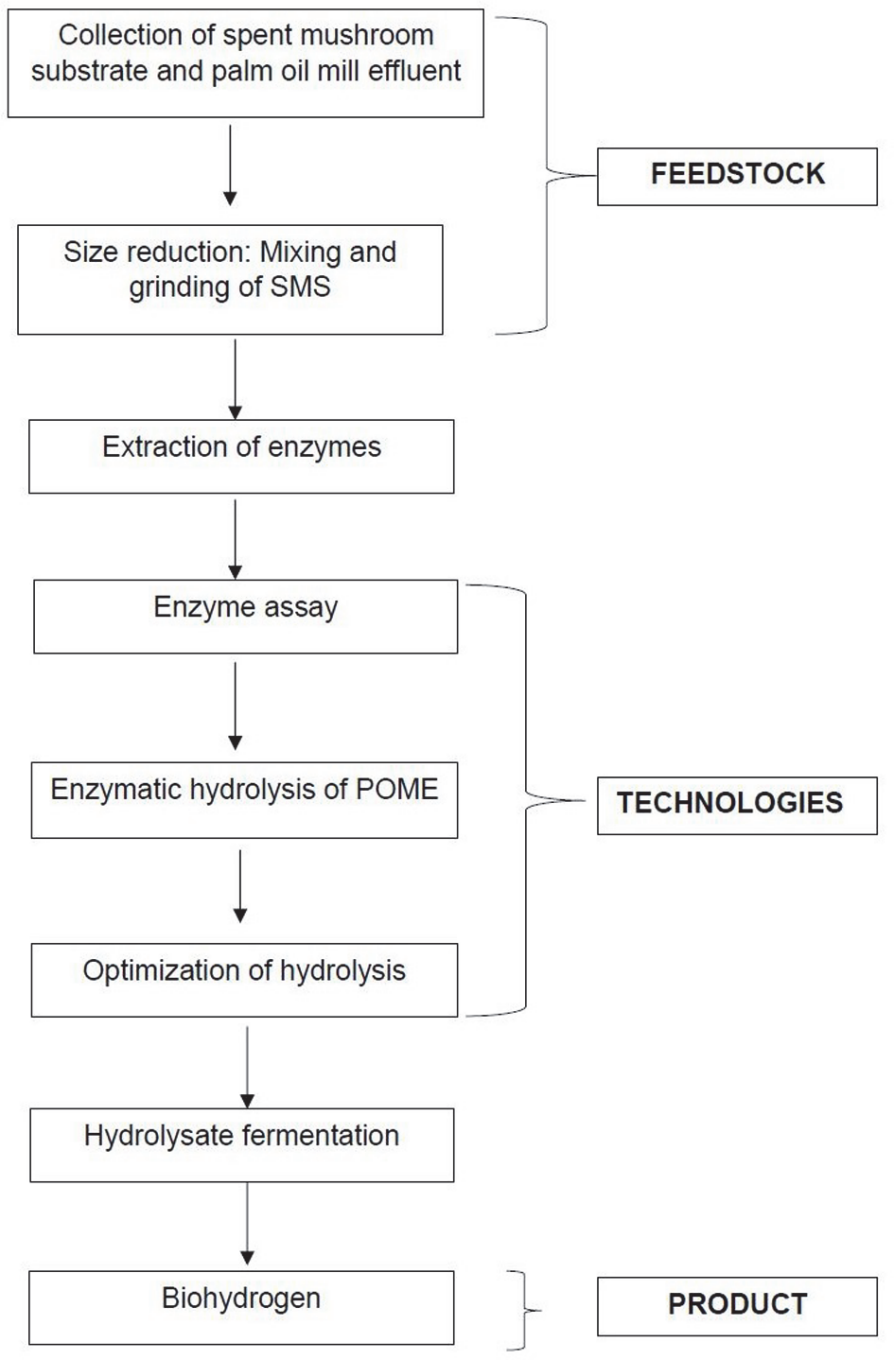
Process flow for the hydrolysis of POME.

Batch fermentation for biohydrogen production was conducted according to Khaleb *et al*. [19] with modification. The POME sludge was used as inoculum for the hydrogen fermentation process with the pre-settled POME as the control and the hydrolyzed POME as the substrate. The study batch was conducted by a working amount of 100 ml using 156 ml serum bottles [33]. The fermentation process was started by adding 20% (v/v) of POME sludge to 80% (v/v) of the POME hydrolysate. Following that, an anaerobic condition was created by sparging nitrogen gas for 10 min during the start-up of the fermentation. The fermentation process was performed in a mesophilic condition (37°C) with an initial pH of 5.5 and agitation maintained at 150 rpm for 24 h. The gas chromatography (Model Perkin Elmer, Autosystem GC) was provided with a thermal conductivity detector (TCD) and a packed GC column (Supelco with 40/80 carboxen 1000, MR2924D; 10’ × 18’) was utilized to quantify the biogas composition [32]. Fig. 1 shows the process flow from enzyme extraction to the batch biohydrogen production in this study.

## Results and Discussions

### Analysis of Enzyme Assays

Cultivated *P. pulmonarius* has two phases of production growth (mycelium and fruiting body stage). During the vegetative stage, the mycelia secrete enzymes to degrade celluloses and lignin in lignocellulosic biomass. During the reproductive stage, the fruiting body’s formation generally lasts for two to three days superseded by a resting phase of about ten days before the second cycle of the fruiting stage. Generally, the *P. pulmonarius* fruit bodies were harvested over five to six cycles before the mushroom bags were deposited as agricultural wastes. The SMS from the eleventh week was selected based on the enzyme profiles (unpublished data for the enzyme profiles from the first week to tenth week of SMS). Enzyme profiles of this study illustrated a periodical bell-shaped pattern of all the enzyme productivities of *P. pulmonarius*, comparable to other white rot fungi such as *Pleurotus ostreatus* [34] and *Grifola frondosa* [35].

The enzyme activities and productivities of the SMS were measured and presented in Table 1. β-glucosidase productivity in this study (915.39 U/g) was 10.2-folds higher than the β-glucosidase productivity of *Pleurotus sajor-caju* after twelve weeks’ inoculation into mushroom bags (89.73 U/g) reported by Singh *et al*. [36]. This could be due to the use of concentrated enzymes in this study as compared to the study by Singh *et al*. [36], which used crude enzyme extract. Meanwhile, laccase productivity in this study (4.41 U/g) was comparable to laccase productivity of SMS (3.0 U/g) extracted from *P. pulmonarius* after the second harvest in a study reported by Ariff *et al*. [13]. This could be an indication of mycelia accumulation during the vegetative stage of growth.

The hydrolysis of cellulose is restricted due to the high cost of commercial cellulase enzymes [37]. Normally, the price of commercial enzymes constitutes about 80% of the final cost of hydrolysis of agroresidues to fermentable sugars [38]. Since the management of SMS in bulk will require high storage and transportation costs, mushroom growers might do well to consider their SMS as valuable feedstocks for conversion or extraction into value-added products [39, 40]. Efficient hydrolysis strategies have been developed to increase enzyme production while reducing the operation cost [41].

Hence, this study showed that SMS of *P. pulmonarius* could be a good source of extracellular enzymes but requires an economical method of extracting the enzymes from the SMS. However, enzyme extraction on a large scale might not be feasible economically if it requires a cold environment for one hour. Application of a homogenizer at room temperature can be an alternative for extraction as the enzymes were able to diffuse into the extraction medium within a shorter time. Thus, the pH tolerance and temperature tolerance of the five enzymes in this study were not determined as the main objective here was to test the feasibility of using enzymes extracted from locally collected SMS for the hydrolysis of POME.

### Optimization of Enzymatic Hydrolysis

POME contains a large amount of insoluble suspended solids and organic matter. POME has potential as a substrate for generating bioenergy, especially biogas due to high organic compounds [42]. Since POME is readily available from the mills, it could served as best source for renewable energy. Therefore, an optimum level of biodegradable sugars from the POME could be obtained with a suitable hydrolysis step [43]. RSM analysis could be applied as it provides visual responses for interaction among the experimental factors [44]. Furthermore, a matrix of CCD in RSM requires only a minimal number of experiments while yielding systematic results, and less reagent usage [45]. According to Neoh *et al*. [46], CCD consists of a two-level factorial design with center and axial points that would provide the optimum result over a specified range of variables. The matrix for the CCD and outcomes for the optimization of reducing sugar yield are displayed in Table 2. The highest reducing sugar yield (3.82 g/l) was obtained from hydrolysis using 10% enzyme loading at pH 5.4 and the lowest sugar yield (0.90 g/l) was obtained from hydrolysis without enzyme added to the POME. Khaw and Ariff [18] suggested that an increase in saccharification rate and sugar yield was found proportional to increasing enzyme concentration.

Results acquired from the optimization experiments were analyzed using ANOVA (Table 3). The model’s *F*-value of 38.41 demonstrated that the model was significant (*p* < 0.0001) and there was 0.01% chance that the model’s *F*-value might arise considering noise. The *p*-value for the models A, B, and A^2^ were significant, *p* < 0.0500. The model is applicable and could be approved since the coefficient of determination (R^2^) of the model was 0.9719, which conveys model competency.

Equation 2 comprises 1 offset term, 3 linear terms, 3 quadratic terms and 3 interactions, represents the quadratic model for the response. The equation shows a valid correlation amongst the parameters studied and the data acquired appeared well-suited to the second-order polynomial in comparison to other models:

$$Y(g/l)=2.79+0.23A+1.10B-0.039C-0.60A^{2}-0.22B^{2}-4.545E-003C^{2}+0.15AB+0.074AC-0.024BC$$ (2)

where the coded variable Y symbolizes concentration of the reducing sugar, whilst A, B, and C represent time, enzyme loading and pH respectively.

**Fig. 2:**
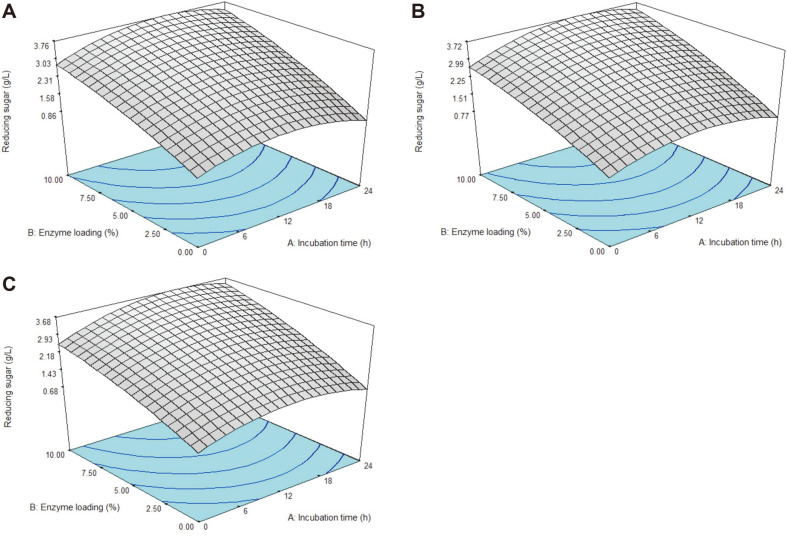
Response surface plot of reducing sugar yield representing the effect of incubation time and enzyme loading. (**A**) pH 4.8. (**B**) pH 5.4. (**C**) pH 6.0.

The 3D contour plots of the model for variation in reducing sugar yield, as a function of time (A) and enzyme loading (B) at three different pH values (4.8, 5.4, and 6.0) was shown in Fig. 2. From Fig. 2A, an increase of reducing sugar concentration would be acquired with increase of the incubation time until 12 h while increasing enzyme loading from 0 to 10% (v/v). The hydrolysis was characterized by an initial logarithmic stage indicating the rapid production of reducing sugars [18]. A highest reducing sugar concentration of 3.76 g/l would be acquired at pH value 4.8 when a high amount of enzyme loading of 10% was used with the addition of incubation time of more than 12 h. However, an extended incubation time of more than 18 h would lead to decrease in reducing sugar yield to 3.03 g/l, which might be due to the presence of cellulase inhibitors that prevent further cellulose conversion into sugar [47].

A total of 3.72 g/l reducing sugar would be acquired when pH 5.4 was used at the center point values between 15 h and 10% of enzyme loading (v/v) (Fig. 2B). As the pH value increased to pH 6.0, 3.68 g/l reducing sugar was achieved at the high amount of enzyme loading (10%) and between 12 to 18 h incubation time (Fig. 2C). Certain factors were recorded that could affect enzymatic hydrolysis of the POME, concurrently reducing sugar yield. Zhu *et al*. [48] explained that the structural features of substrates, solids loading, enzyme loading, and hydrolysis period greatly affected the rate of enzymatic hydrolysis.

**Fig. 3:**
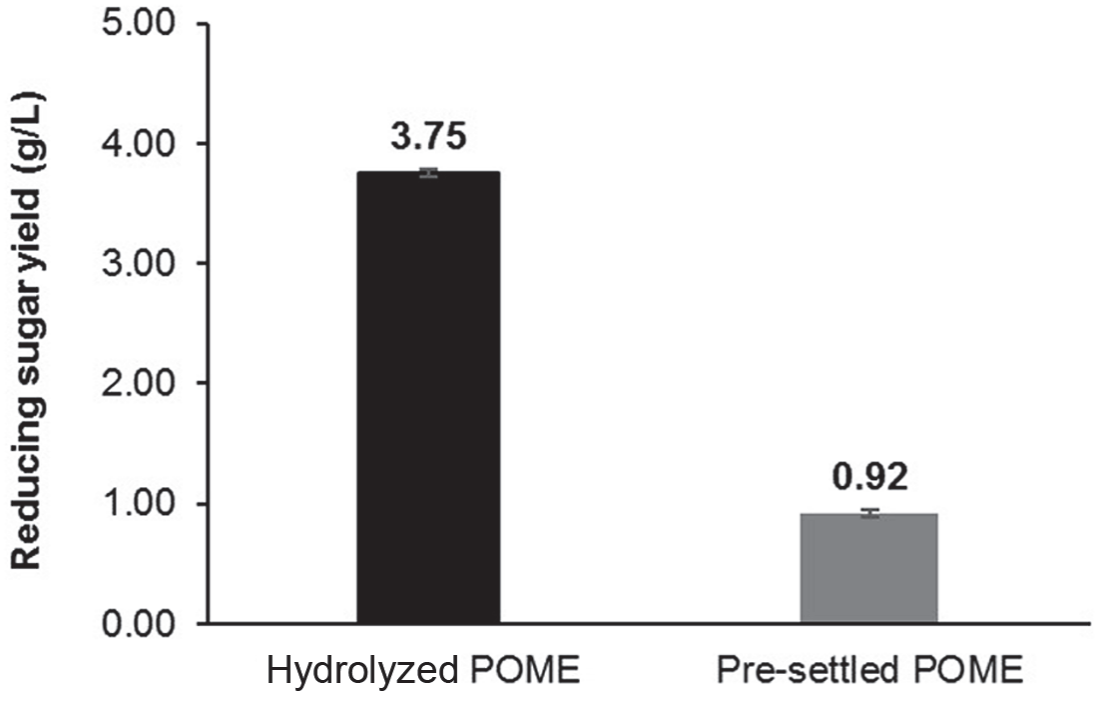
Reducing sugar yield of hydrolyzed POME and pre-settled POME in the verification experiment.

Mun *et al*. [49] recorded a total of 22.8 g/l reducing sugar when the POME solid was hydrolyzed at 12 h using mixed cellulase enzymes (Table 4). The sugar yield by Mun *et al*. [49] was higher compared to this study probably due to the pretreatment of POME solid using sulfuric acid. The dilute sulfuric acid pretreatment of lignocellulosic biomass could increase the access to the substrate by enzymes [50]. Khaw and Ariff [18] recorded a high production of reducing sugar from hydrolysis of POME solid (9.26 g/l) when a Novozyme/Celluclast ratio of 0.60 was used, probably due to the high proportion of cellulase enzyme combination and enzyme dosage. Meanwhile, Silvamany *et al*. [16] reported higher reducing sugar yield after hydrolysis of POME liquid compared to this study possibly due to the use of commercial cellulase enzymes. Silvamany *et al*. [16] also reported that the reducing sugar from POME hydrolysis remained constant after 24 h indicating the completion of saccharification process. The differences in reducing sugar might be due to the quality of fresh fruit bunches and the efficiency of the machines used during the extraction process. Pandiyan *et al*. [51] discovered enzymatic hydrolysis of alkaline pretreated *Parthenium* sp. released a total reducing sugar of 85.8%. In another study, the hydrolysis of oil palm trunk by crude enzymes from *Aspergillus fumigatus* released 13.15 g/l of reducing sugar [52]. The variations in the yield of reducing sugar were possibly contributed by the variety of chemical content in biomass and pretreatment conditions [53]. The prediction was that the hydrolysis rate of POME would be highest at 15 h of incubation. This might be due to the delignification and decrystallization of substances in POME and the increase in enzymes catalytic activities. As the time extended, the enzyme active sites might have decreased, and thus decrease the hydrolysis rate [54]. An optimum reducing sugar of 3.76 g/l^-1^ was estimated with 15 h of incubation time, at 10%enzyme loading (v/v) and pH value of 4.8. As for the verification of the optimized condition, hydrolysis of POME released 3.75 g/l of total reducing sugar, which was consistent with the estimated value (Fig. 3). Hence, the model was suitable to predict the optimal levels of the experiment variables.

Thus, enzymatic hydrolysis of POME using enzymes from SMS is feasible to produce fermentable sugar considering this process does not requires specialized material in the equipment and can be performed with low energy consumption [18].

### Batch Biohydrogen Production

After 24 h of batch fermentation by POME sludge, a maximum biohydrogen production (302.78 ml H_2_.L^-1^ POME) was recorded from the hydrolyzed POME which was 23-folds higher than the pre-settled POME (13.38 ml H_2_.L^-1^ POME) (Table 5). The low biohydrogen production from the pre-settled POME as compared to hydrolyzed POME might be due to the high metabolite accumulation in the pre-settled POME. However, analysis of soluble metabolites, organic acids produced during hydrolysis, and particular analysis on biohydrogen production during the fermentation process was excluded in this study as these were not the main scope of this study. Nevertheless, the percentage of hydrogen and carbon dioxide produced in this study was comparable to Fang and Liu [55], where the hydrogen content ensued an opposite trend of carbon dioxide at the pH value between 4.0 to 7.0.

Fermentative biohydrogen yield is influenced by several aspects including pH, temperature, substrate concentration and the amount of inoculum feed [56]. The production of biohydrogen in this study was 14-folds lower than the biohydrogen production recorded by Kamal *et al*. [22] where the POME was pre-treated with acid and heat which could be hazardous, poisonous, and risky in a laboratory setting. Moreover, the acid-heat pretreatment method is an expensive process and might have formed inhibitory compounds such as furfural [57]. The biohydrogen production in this study (302.78 ml H_2_.L^-1^ POME) was lower than the biohydrogen production reported by Khaleb *et al*. [19], which was 1,439 ml H_2_.L^-1^ of POME (Table 6). The difference might be due to the use of purified and concentrated commercial enzymes (Celluclast 1.5L and Novozyme 188) during the initial POME hydrolysis. This condition led to a higher degree of sugar degradation and higher biohydrogen production. However, this study provides a more economical way to prepare lignocellulolytic enzymes for POME hydrolysis.

In addition, the biohydrogen production in this study was 2-folds higher compared to the study by Garritano *et al*. [58] that used plant enzyme preparation extracted from dormant castor bean seeds, as well as higher pH (pH 6.5) applied during the fermentation process. Fang and Liu’s [55] study found that the biohydrogen yield hit the maximum value at pH of 5.5. This condition was the same as that of Chong *et al*. [59] where the highest hydrogen output of 3,195 ml H_2_.L^-1^ POME was released at pH of 5.5.

Most hydrogen is generated from fossil fuels as the demand is increasing worldwide. However, fossil fuel energy has caused obnoxious environmental pollution [19]. The alternative for sustainable hydrogen production is through fermentation. The biological process has several advantages including low sludge formation, low energy demand, lack of unpleasant smell and production of methane due to the efficient degradation of organic compounds by bacteria [60].

Recently, many studies have utilized starch or simple sugars via dark fermentation which is costly for biohydrogen production [61-63]. Thus, the new research trend is to utilize cheap and readily available POME for biohydrogen production [14]. The utilization of this lignocellulosic SMS and POME could promote the agricultural economy, reduce greenhouse gas emissions, and improve energy security [64]. In addition, the mushroom industry produces 24 tons of SMS per month [3] and has been reported to contribute approximately£90 million to the economy of the Republic of Ireland [65], while securing 3,000 jobs [66]. Nowadays, dried SMS is sold for more than 10 RMB Yuan/50 kg at Sanzhi District for biogas production [67]. Meanwhile, Najafi *et al*. [68] recorded that one period of SMS production releases biogas energy equal to 277.95 kW.h/ton at 35°C and 322.84 kW.h/ton at 55°C.

Thus, both SMS and POME are suitable resources for producing value-added products. The results of this study warrant a scale-up of biohydrogen production using hydrolyzed POME.

## Conclusion

The disposal of spent mushroom substrate has been a major issue for mushroom growers worldwide. SMS is either discarded in landfills or directly burnt which cause environmental pollution. Thus, the present study identified the potential of using concentrated enzymes extracted from SMS in the hydrolysis of POME. Using response surface methodology, incubation time and enzyme loading factors had significant effects (*p* < 0.05) on the POME hydrolysis. A total of 3.75 g/l reducing sugar was obtained at 15 h incubation time with 10% (v/v) enzyme loading, indicating that the enzymes extracted from SMS could be a renewable resource for hydrolysis of agroresidues. Batch fermentation using hydrolyzed POME (302.78 ml H_2_.L^-1^ POME) for biohydrogen production yielded 23 times higher volume of biohydrogen compared to non-hydrolyzed pre-settled POME (13.38 ml H_2_.L^-1^ POME). The direct utilization of the spent mushroom substrate for enzymatic hydrolysis can be further explored to determine the economics aspects and could be a favorable choice for recovery of biofuel (hydrogen) product. Hydrolysis of POME using enzymes from SMS illustrates a cost-effective upscaling strategy for an economical conversion to reducing sugars.

## Figures and Tables

**Table 1:** Enzyme activities and productivities from SMS of *P. pulmonarius*. The results are the mean of three samples.

Enzymes	Activities (U/ml)	Productivities (U/g)
Lignin Peroxidase	42.82 ± 0.26	214.14 ± 1.31
Laccase	0.82 ± 0.01	4.14 ± 0.05
Xylanase	0.45 ± 0.02	2.25 ± 0.11
Endoglucanase	2.91 ± 0.52	14.56 ± 2.61
β-glucosidase	183.08 ± 10.42	915.39 ± 52.08

**Table 2:** The CCD matrix and optimization results.

Run	Variables			Response

	A: Time (h)	B: Enzyme loading (%, v/v)	C: pH	Reducing sugar (g/l)
1	12	5	5.4	2.91
2	12	5	5.4	2.92
3	12	5	5.4	2.90
4	24	10	6.0	3.44
5	0	0	4.8	0.90
6	24	5	5.4	2.56
7	12	5	4.8	2.79
8	0	10	4.8	2.90
9	12	5	6.0	2.63
10	0	5	5.4	1.66
11	0	10	6.0	2.49
12	12	5	5.4	2.86
13	24	0	4.8	0.97
14	12	10	5.4	3.82
15	12	5	5.4	2.89
16	12	5	5.4	2.55
17	0	0	6.0	0.90
18	24	0	6.0	0.95
19	24	10	4.8	3.24
20	12	0	5.4	1.16

**Table 3:** ANOVA acquired from the optimization hydrolysis of POME.

Source	Sum of square	DF	Mean square	F value	Prob > F	Remarks
Model	15.81	9	1.76	38.41	< 0.0001	Significant
A	0.53	1	0.53	11.67	0.0066	Significant
B	12.12	1	12.12	265.09	< 0.0001	Significant
C	0.02	1	0.02	0.33	0.5769	Not significant
A^2^	1.01	1	1.01	21.98	0.0009	Significant
B^2^	0.14	1	0.14	3.03	0.1122	Not significant
C^2^	5.682E-005	1	5.682E-005	1.243E-003	0.9726	Not significant
AB	0.17	1	0.17	3.74	0.0818	Not significant
AC	0.04	1	0.04	0.95	0.3523	Not significant
BC	4.513E-003	1	4.513E-003	0.10	0.7599	Not significant
Residual	0.46	10	0.05			
Lack of fit	0.36	5	0.07	3.49	0.0983	Not significant
Pure error	0.10	5	0.02			
Total	16.26	19				

SD	0.21	C.V.	9.02	R^2^	0.9719	
Mean	2.37	PRESS	3.48	Adj. R^2^	0.9466	
				Pred. R^2^	0.7858	
				Adeq. Prec.	19.740	

*DF: degree of freedom; SD: standard deviation; C.V.: coefficient of variation; PRESS: prediction error sum of squares; Adj. R^2^: adjusted R-squared; Pred. R^2^: predicted R-squared; Adeq. Precision: adequate precision

**Table 4:** Comparison of sugar yield after enzymatic hydrolysis of POME.

Enzyme	POME	Pretreatment	Condition	Sugar yield (g/L)	Reference
Crude mixture from *Aspergillus niger* EB5 and *Trichoderma* sp. EB6	Solid	0.5% (v/v) sulfuric acid	12 h 50 °C pH 5.0	22.80	[48]
Combination of Celluclast 1.5L and Novozyme 188	Solid	None	6 h 40 °C pH 5.0	9.26	[18]
Combination of Celluclast 1.5L, Novozyme 188 and Viscozyme-L	Liquid	None	48 h 50 °C pH 4.8	Centrifugal waste: 34.30 Sterilizer condensate: 6.50	[16]
Crude enzymes from spent mushroom substrate	Liquid	None	12 h 50 °C pH 5.4	3.82	This study

**Table 5:** Outcomes of POME composition after 24 h of batch fermentation.

POME composition	Hydrolyzed POME^*^	Pre-settled POME^*^
Hydrogen cumulative volume (ml H_2_.L^-1^ POME)	302.78 ± 13.57	13.38 ± 3.36
Hydrogen productivity (ml H_2_.L^-1^ POME h^-1^)	12.62 ± 0.57	0.56 ± 0.14
Percentage of hydrogen, H_2_ (%)	83.52 ± 2.37	36.35 ± 8.17
Percentage of carbon dioxide, CO_2_ (%)	16.48 ± 2.37	63.65 ± 8.17
Hydrogen volume (ml)	24.22 ± 1.09	1.07 ± 0.27
Hydrogen production rate (ml H_2_.h^-1^)	1.01 ± 0.05	0.04 ± 0.01

*Values represent means of three replicate samples.

**Table 6:** Comparison in biohydrogen production after fermentation employing POME as a substrate.

Temp. (°C), pH	Treatment of POME	Type of microorganisms	Biohydrogen production (mL H_2_/ L POME)	Biohydrogen productivity (mL H_2_/ L POME/ h)	Reference
37, 7.0	Acid-heat treatment	Bacteria	4304.00	NA	[22]
37, 6.0	Enzymatic hydrolysis	Fungal enzyme	1439.00	87.19	[19]
35, 6.5	Enzymatic hydrolysis	Plant enzyme	134.74	8.42	[57]
37, 5.5	NA Bacteria	3195.00	1034	[58]
37, 5.5	Enzymatic hydrolysis	Fungal enzyme	302.78	12.62	This study

NA: not available
